# Supplementary material for: Reconfigurable self-assembly of photocatalytic magnetic microrobots for water purification
Source: Nat Commun. 2023 Nov 1;14:6969. doi: 10.1038/s41467-023-42674-9 (PMC10620202; doi:10.1038/s41467-023-42674-9)
Supplement: Supplementary file 1 — Supplementary Information [file 41467_2023_42674_MOESM1_ESM.pdf]

Reconfigurable self-assembly of photocatalytic magnetic microrobots for water purification

Mario Urso[1], Martina Ussia[1], Xia Peng[1], Cagatay M. Oral[1], Martin Pumera[1,2,3,4*]

[1] Future Energy and Innovation Laboratory, Central European Institute of Technology, Brno University of Technology, Purkyňova 123, 61200 Brno, Czech Republic

[2] Faculty of Electrical Engineering and Computer Science, VSB - Technical University of Ostrava, 17. listopadu 2172/15, 70800 Ostrava, Czech Republic

[3] Department of Medical Research, China Medical University Hospital, China Medical University, Hsueh-Shih Road 91, 40402Taichung, Taiwan

[4] Department of Chemical and Biomolecular Engineering, Yonsei University, Yonsei–ro 50, Seodaemun–gu, 03722 Seoul, Republic of Korea

E-mail: pumera.research@gmail.com

Supplementary Information

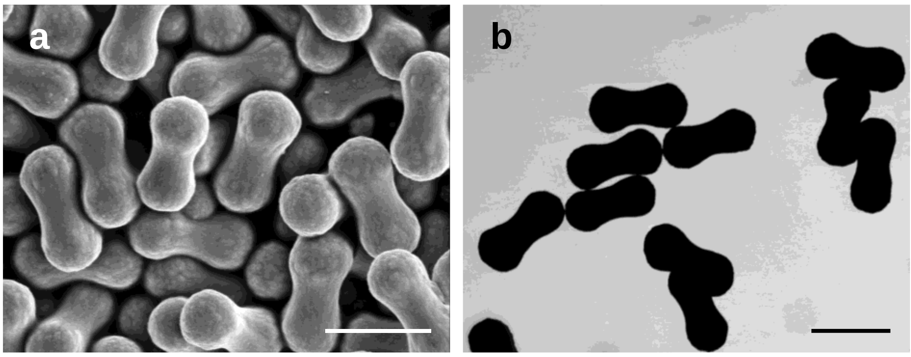

Supplementary Fig. 1: Characterization of TiO_2/α-Fe_2O_3 microrobots by SEM and STEM analyses. a SEM and **b** STEM images of TiO_2/α-Fe_2O_3 microrobots. Scale bars are 2 µm.

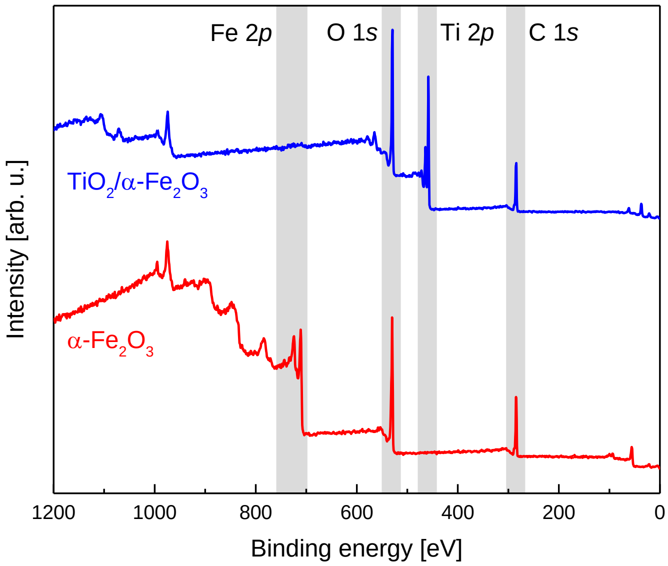

Supplementary Fig. 2: Characterization of samples by XPS analysis. XPS wide spectra of α-Fe_2O_3 microparticles and TiO_2/α-Fe_2O_3 microrobots.

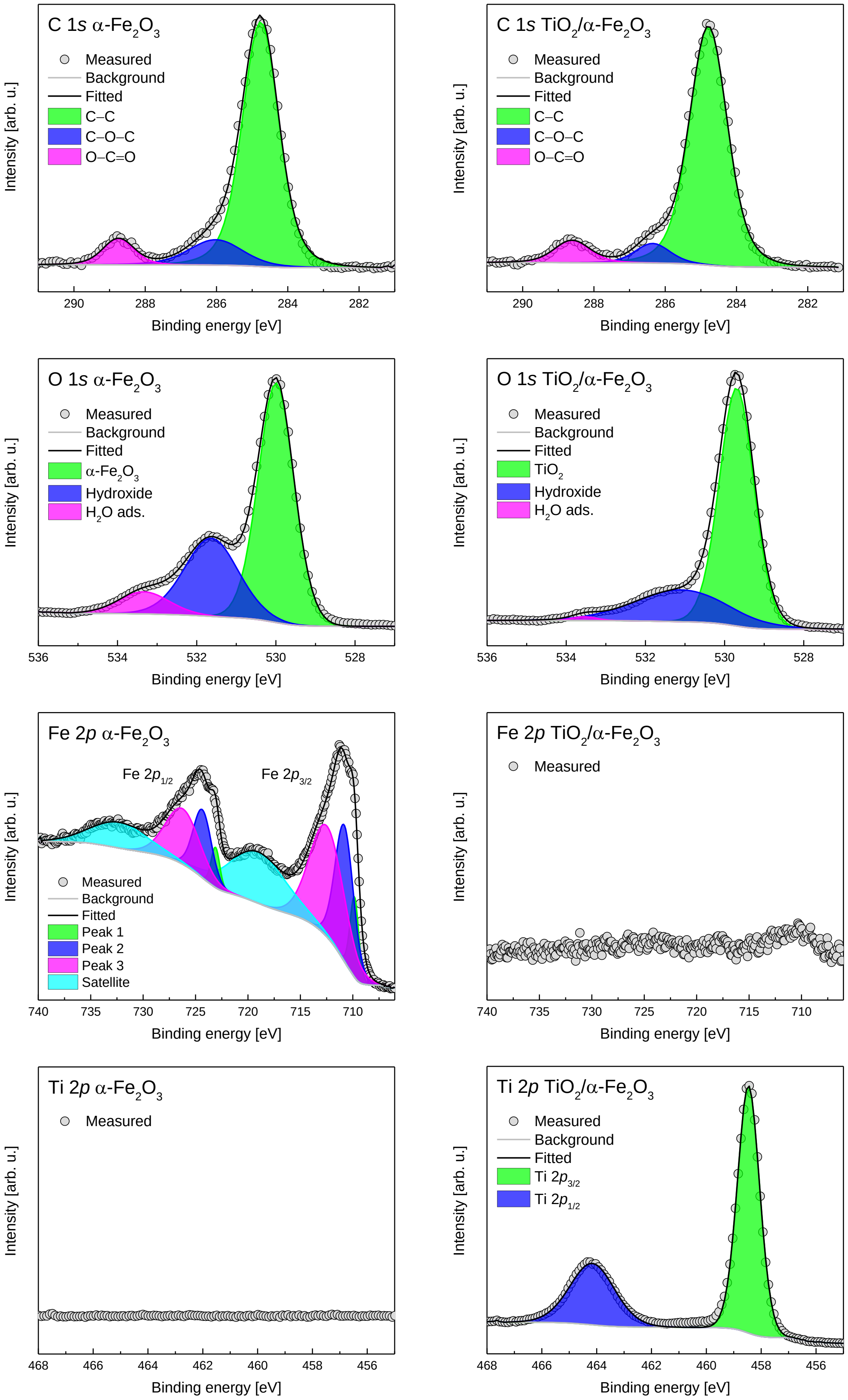

Supplementary Fig. 3: Characterization of samples by high-resolution XPS analysis. High-resolution XPS spectra of C 1*s*, O 1*s*, Fe 2*p*, and Ti 2*p* for α-Fe_2O_3 microparticles and TiO_2/α-Fe_2O_3 microrobots.

Supplementary Table 1. XPS peak fitting results for α-Fe_2O_3 microparticles and TiO_2/α-Fe_2O_3 microrobots.

Sample	Region	Binding energy [eV]	Assigned to	Ref.
α-Fe_2O_3	C 1*s*	284.8<br>286.0<br>288.8	C–C<br>C–O–C<br>O–C=O	1
	O 1*s*	530.0<br>531.6<br>533.3	α-Fe_2O_3<br>Hydroxide<br>H_2O ads.	2
	Fe 2$p_{3/2}$<br>(Fe 2$p_{1/2}$)	709.8 (723.1)<br>710.8 (724.4)<br>712.5 (726.2)<br>719.1 (732.3)	Peak 1<br>Peak 2<br>Peak 3<br>Satellite	2
TiO_2/α-Fe_2O_3	C 1*s*	284.8<br>286.4<br>288.6	C–C<br>C–O–C<br>O–C=O	1
	O 1*s*	529.7<br>531.1<br>533.5	TiO_2<br>Hydroxide<br>H_2O ads.	3
	Ti 2*p*	458.5<br>464.2	Ti 2$p_{3/2}$<br>Ti 2$p_{1/2}$	3

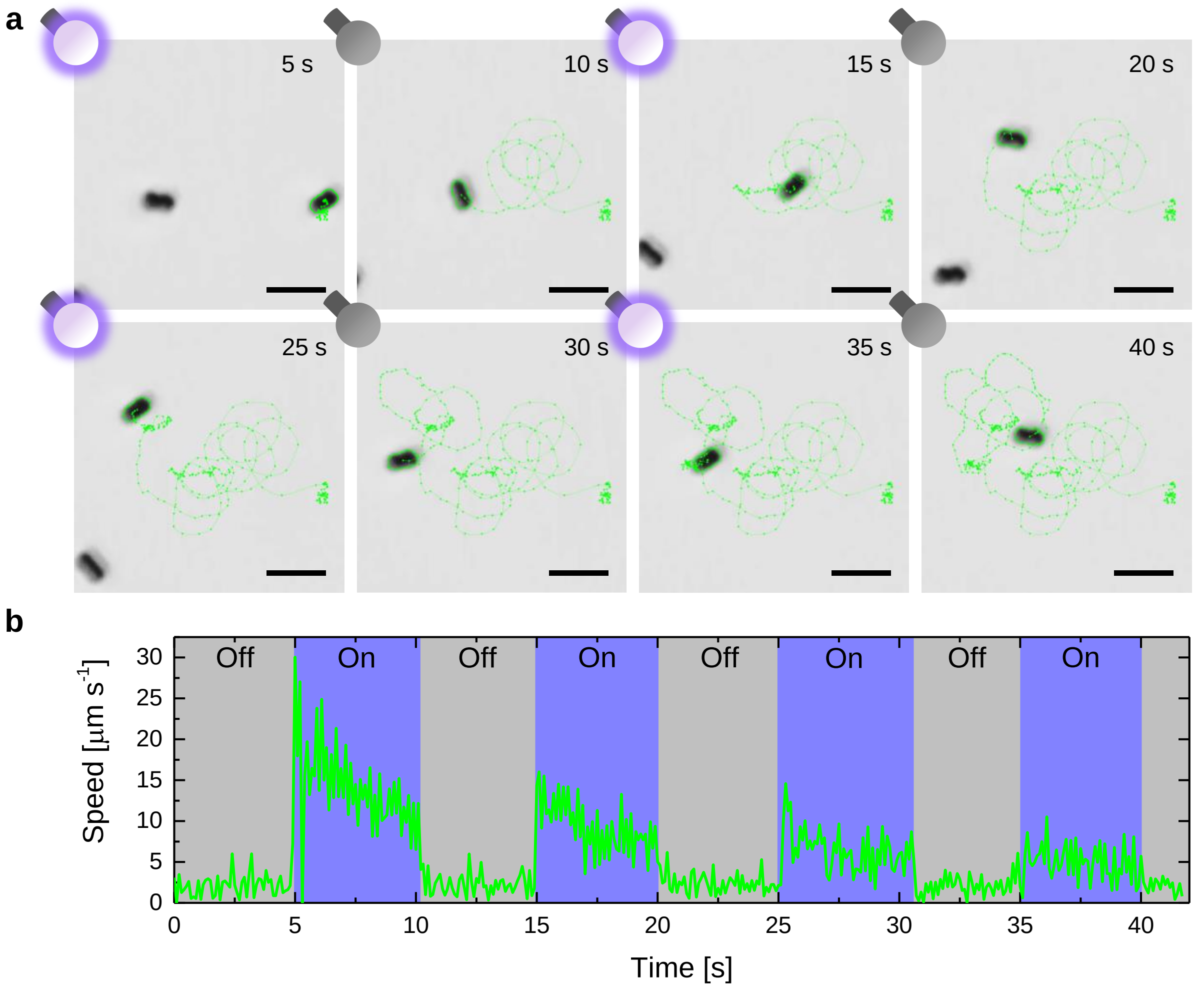

Supplementary Fig. 4: Influence of the on/off switching of light on the motion behavior of TiO_2/α-Fe_2O_3 microrobots. a Time-lapse micrographs showing the trajectory of a TiO_2/α-Fe_2O_3 microrobot at 5 s on/off switching of UV light irradiation in pure water and **b** the corresponding instantaneous speed as a function of time. Scale bars are 5 µm.

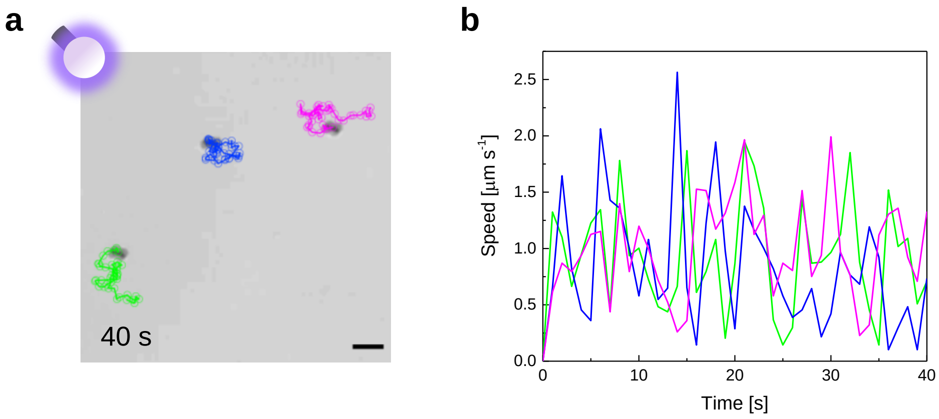

Supplementary Fig. 5: Motion behavior of α-Fe_2O_3 microparticles. a Micrograph showing the trajectories of three α-Fe_2O_3 microparticles under UV light irradiation in pure water for 40 s and **b** the corresponding instantaneous speed as a function of time. The scale bar is 5 µm.

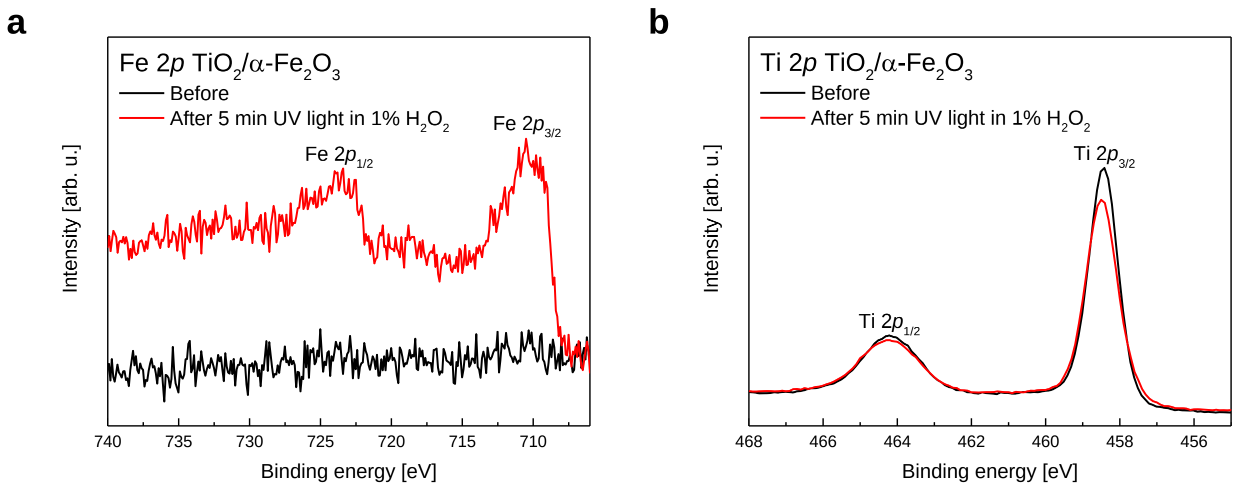

Supplementary Fig. 6: Evidence of the partial corrosion of the TiO_2 layer of TiO_2/α-Fe_2O_3 microrobots by XPS analysis. High-resolution XPS spectra of **a** Fe 2*p* and **b** Ti 2*p* for TiO_2/α-Fe_2O_3 microrobots before and after exposure to UV light irradiation in 1% H_2O_2 for 5 min.

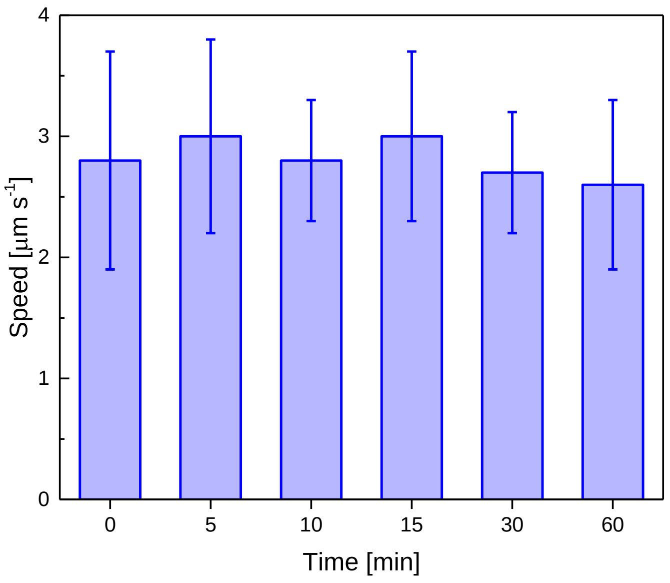

Supplementary Fig. 7: Lifetime of the speed of TiO_2/α-Fe_2O_3 microrobots. Speed of TiO_2/α-Fe_2O_3 microrobots under UV light irradiation in pure water as a function of time. Error bars represent the standard deviation, $n = 20$ independent microrobots.

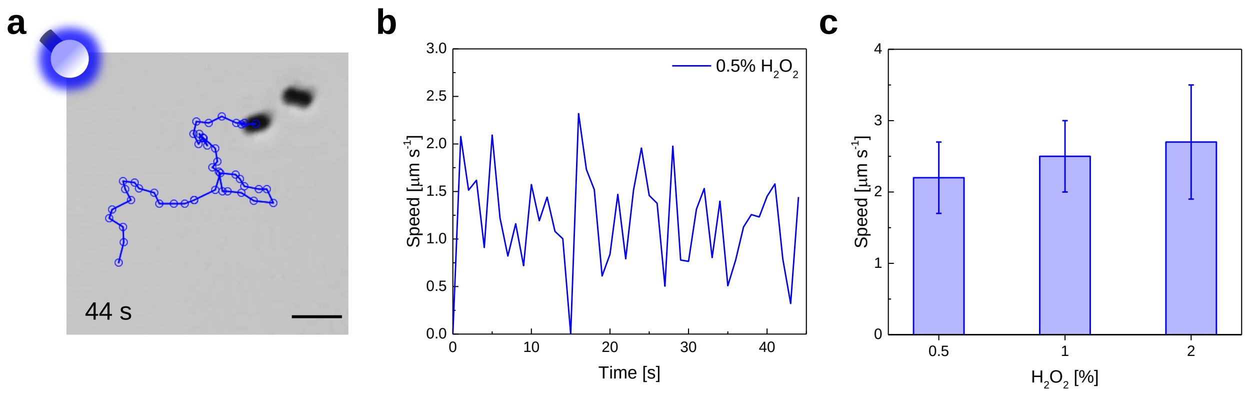

Supplementary Fig. 8: Motion behavior of TiO_2/α-Fe_2O_3 microrobots under visible light irradiation. a Micrograph showing the trajectory of a TiO_2/α-Fe_2O_3 microrobot under blue light irradiation in 0.5% H_2O_2 for 44 s and **b** the corresponding instantaneous speed as a function of time. The scale bar is 5 µm. **c** Microrobots' speed as a function of H_2O_2 concentration under blue light irradiation. Error bars represent the standard deviation, $n = 20$ independent microrobots.

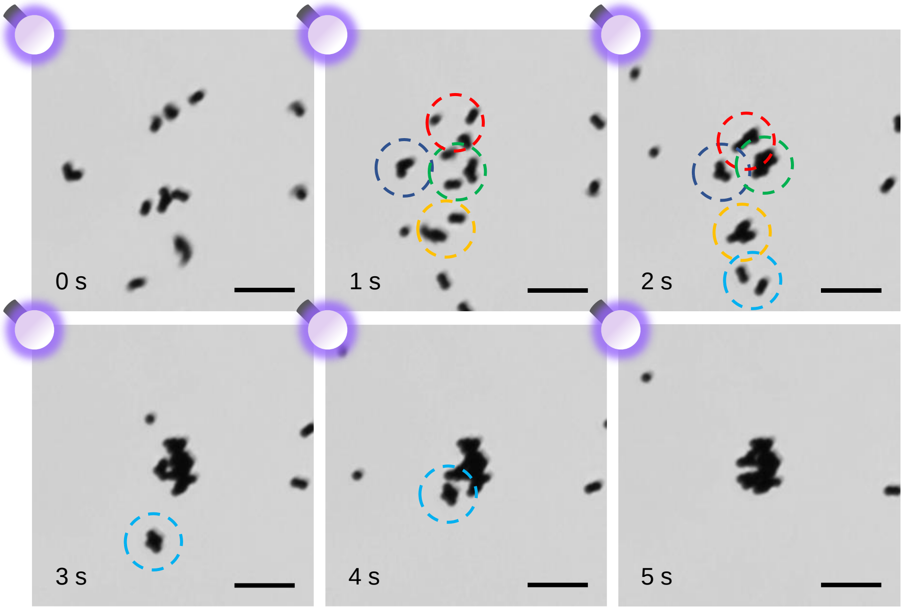

Supplementary Fig. 9: Self-assembly of TiO_2/α-Fe_2O_3 microrobots into a cluster. Time-lapse micrographs showing the clustering process of TiO_2/α-Fe_2O_3 microrobots under UV light irradiation in 1% H_2O_2 for 5 s. Scale bars are 10 µm.

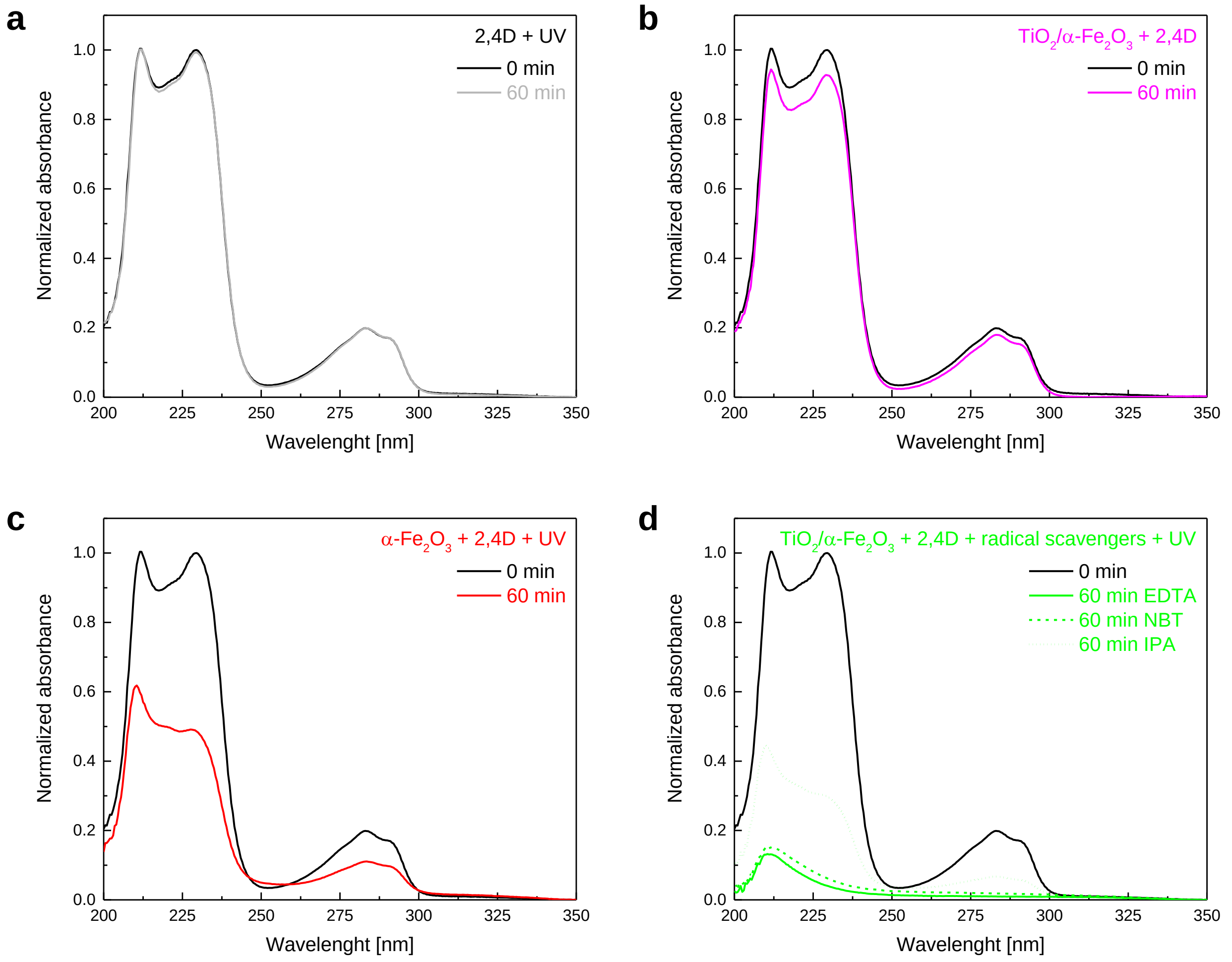

Supplementary Fig. 10: Absorbance spectra of 2,4D solutions subjected to different treatments. Absorbance spectra of 2,4D solutions (5×10^{-5} M) before (0 min) and after the treatment with **a** UV light irradiation in pure water for 60 min, **b** TiO_2/α-Fe_2O_3 microrobots (1 mg mL^{-1}) without UV light irradiation in pure water for 60 min ("no motion" condition), **c** α-Fe_2O_3 microparticles (1 mg mL^{-1}) and UV light irradiation in pure water for 60 min, **d** TiO_2/α-Fe_2O_3 microrobots (1 mg mL^{-1}), a radical scavenger (10 mg L^{-1} EDTA, 10 mg L^{-1} NBT, or 0.25 µL mL^{-1} isopropanol), and UV light irradiation in pure water for 60 min.

Supplementary Discussion 1

H_2O_2 consumption experiments were performed to measure the H_2O_2 consumption rate by TiO_2/α-Fe_2O_3 microrobots under UV light irradiation. For this purpose, UV-transparent cuvettes were filled with 1 mL of solution containing microrobots (1 mg mL^{-1}) and 1% H_2O_2, and exposed to UV light irradiation for different durations (0, 10, 30, and 60 min). Afterward, microrobots were separated by centrifugation to record the UV-Vis absorbance spectra of treated solutions. The H_2O_2 concentration was determined from absorbance spectra according to a previous work.[4] Supplementary Fig. 11 shows the time dependence of the H_2O_2 concentration. The slope of the linear fit in the range 0÷30 min gave an H_2O_2 consumption rate of 0.011 mol L^{-1} min^{-1}, corresponding to 1.8×10^{-7} mol s^{-1} for 1 mL of solution. To normalize the rate per unit area [mol s^{-1} m^{-2}], TiO_2/α-Fe_2O_3 microrobots' surface area was estimated. For simplicity, the microrobots were assumed to be peanut-shaped α-Fe_2O_3 microparticles consisting of two adjacent spheres with a radius of 0.56 µm, as measured by STEM analysis. Considering that the volume (V) of a spherical particle with radius r is $V = \frac{4}{3}\,\pi\, r^3$, the volume occupied by a microrobot was found as ~1.5×10^{-18} m^3. Given an α-Fe_2O_3 density (ρ) of 5.3 g cm^{-3}, the mass (m) of the single microrobot was calculated to be 8.0×10^{-12} g through the relation $m = \rho\, V$. Therefore, in 1 mL of solution with a concentration of microrobots of 1 mg mL^{-1}, there are 1.3×10^{8} microrobots. Since the surface (A) of a spherical particle with radius r is $A = 4\,\pi\, r^2$, the area exposed by a microrobot was calculated to be 7.9×10^{-12} m^2, whereas the surface area of 1 mg of microrobots was found as 1.0×10^{-3} m^2. Consequently, the surface area-normalized H_2O_2 consumption rate was calculated to be approximately -1.8×10^{-4} mol s^{-1} m^{-2}.

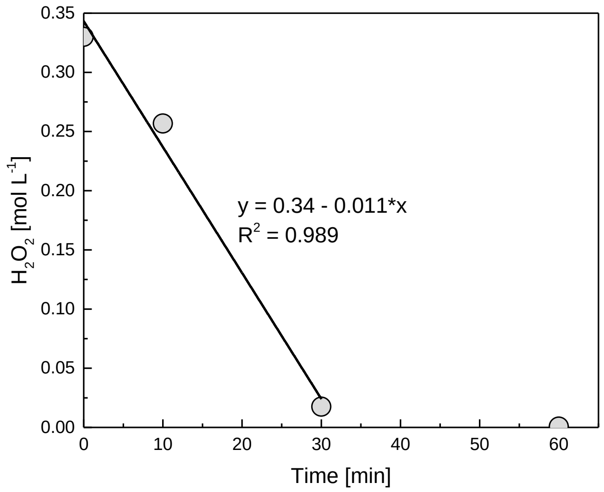

Supplementary Fig. 11: H_2O_2 consumption experiments. H_2O_2 concentration as a function of time following H_2O_2 consumption experiments by TiO_2/α-Fe_2O_3 microrobots under UV light irradiation in 1% H_2O_2.

Supplementary References

1. Urso, M., Ussia, M., Novotný, F. & Pumera, M. Trapping and detecting nanoplastics by MXene-derived oxide microrobots. *Nat. Commun.* **13**, 3573 (2022).

2. Biesinger, M. C. *et al.* Resolving surface chemical states in XPS analysis of first row transition metals, oxides and hydroxides: Cr, Mn, Fe, Co and Ni. *Appl. Surf. Sci.* **257**, 2717–2730 (2011).

3. Biesinger, M. C., Lau, L. W. M., Gerson, A. R. & Smart, R. S. C. Resolving surface chemical states in XPS analysis of first row transition metals, oxides and hydroxides: Sc, Ti, V, Cu and Zn. *Appl. Surf. Sci.* **257**, 887–898 (2010).

4. Aye, T. T., Low, T. Y. & Sze, S. K. Nanosecond laser-induced photochemical oxidation method for protein surface mapping with mass spectrometry. *Anal. Chem.* **77**, 5814–5822 (2005).
