# Supplementary material for: Reconfigurable self-assembly of photocatalytic magnetic microrobots for water purification
Source: Nat Commun. 2023 Nov 1;14:6969. doi: 10.1038/s41467-023-42674-9 (PMC10620202; doi:10.1038/s41467-023-42674-9)
Supplement: Supplementary file 3 — Description of Additional Supplementary Files [file 41467_2023_42674_MOESM3_ESM.pdf]

Description of Additional Supplementary Files

File Name: Supplementary Movie 1
Description: Microrobots at 5 s on/off switching of UV light irradiation in pure water.

File Name: Supplementary Movie 2
Description: Microrobots under UV light irradiation in pure water.

File Name: Supplementary Movie 3
Description: Magnetic navigation of microrobots under UV light irradiation in pure water.

File Name: Supplementary Movie 4
Description: Reconfigurable, reversible, and active self-assembly of microrobots at 60 s on/off switching of UV light irradiation in 1% hydrogen peroxide.

File Name: Supplementary Movie 5
Description: Microrobot cluster under UV light irradiation in 1% hydrogen peroxide.
